# *Period 2 *regulates neural stem/progenitor cell proliferation in the adult hippocampus

**DOI:** 10.1186/1471-2202-10-30

**Published:** 2009-03-27

**Authors:** Laurence Borgs, Pierre Beukelaers, Renaud Vandenbosch, Laurent Nguyen, Gustave Moonen, Pierre Maquet, Urs Albrecht, Shibeshih Belachew, Brigitte Malgrange

**Affiliations:** 1 Developmental Neurobiology Unit, Center for Cellular and Molecular Neurobiology, University of Liège, C.H.U. B36, 4000 Liège, Belgium; 2 Department of Neurology, C.H.U. Sart Tilman, B35, 4000 Liège, Belgium; 3 Cyclotron Research Center, University of Liège, B30, 4000 Liège, Belgium; 4 Department of Medicine, Division of Biochemistry, University of Fribourg, Friboug, Switzerland

## Abstract

**Background:**

Newborn granule neurons are generated from proliferating neural stem/progenitor cells and integrated into mature synaptic networks in the adult dentate gyrus of the hippocampus. Since light/dark variations of the mitotic index and DNA synthesis occur in many tissues, we wanted to unravel the role of the clock-controlled *Period2 *gene (*mPer2*) in timing cell cycle kinetics and neurogenesis in the adult DG.

**Results:**

In contrast to the suprachiasmatic nucleus, we observed a non-rhythmic constitutive expression of mPER2 in the dentate gyrus. We provide evidence that mPER2 is expressed in proliferating neural stem/progenitor cells (NPCs) and persists in early post-mitotic and mature newborn neurons from the adult DG. In vitro and in vivo analysis of a mouse line mutant in the *mPer2 *gene (*Per2*^*Brdm1*^), revealed a higher density of dividing NPCs together with an increased number of immature newborn neurons populating the DG. However, we showed that the lack of *mPer2 *does not change the total amount of mature adult-generated hippocampal neurons, because of a compensatory increase in neuronal cell death.

**Conclusion:**

Taken together, these data demonstrated a functional link between the constitutive expression of mPER2 and the intrinsic control of neural stem/progenitor cells proliferation, cell death and neurogenesis in the dentate gyrus of adult mice.

## Background

Recent findings have shed light on the integration of adult-born hippocampal dentate gyrus (DG) neurons into mature synaptic networks [1,2]. How and why these new neurons are formed has become an intriguing question [3]. Because the circadian clock machinery can control intrinsic regulation of cell proliferation in peripheral tissues [4] we hypothesized that clock genes might influence neural stem/progenitor cell (NPC) division in the central nervous system.

Adult hippocampal neurogenesis in mammals is a plastic process placed under the control of environmental stimuli, which is comparable to the regulation of circadian rhythms [5]. Hormonal cycles and psychosocial stress [6,7], serotonin metabolism [8], depression [9], aging [10,11], physical activity [12], sleep deprivation [13] and enriched living conditions [14] influence the rate of neuronal renewal and survival in various adult organisms. This suggests that mechanisms controlling life-long neurogenesis in the postnatal CNS are adapted to complex extrinsic inputs. Neurogenesis appears to be regulated by such physiological and behavioural parameters that are somehow connected to the circadian clock that synchronizes itself to changing environmental conditions to optimize an organism's performance. Recent work has consistently shown a diurnal rhythm of neurogenesis among the olfactory projection neurons in the crustacean brain, with peak of neuroblasts proliferation during the hours surrounding dusk, the most active period for lobsters [15]. These data suggest the possibility that light-controlled rhythms may be primary regulators of neuronal proliferation, and that previously demonstrated hormonal and activity-driven influences over adult neurogenesis may be secondary events in a complex circadian control pathway.

Intriguingly, the expression of circadian genes that belong to the intracellular clock of the suprachiasmatic nucleus (SCN) have also been described in other brain areas, in peripheral organs and even in immortalized cell lines in culture [16,17]. The *mPer2 *gene is one of the three mammalian orthologs (*mPer1*, *mPer2 *and *mPer3*) of the Drosophila circadian clock gene *period *[18,19]. In situ hybridization experiments have established that *mPer1 *and *mPer2 *genes are expressed in the hippocampus and particularly in the DG where their function remains elusive [18]. The rate of cell cycle kinetics and neurogenesis are tightly coupled in the adult mammalian DG. The overall duration of the cell cycle in hippocampal neuronal precursor cells is about 24 hours [20] and very close if not identical to the circadian period. There is already substantial in vivo evidence that outside the mammalian brain, circadian rhythms control the timing of cell division [4]. Accordingly, light/dark variations of the mitotic index and DNA synthesis occur in many tissues, i.e. oral mucosa [21], tongue keratinocytes [22], liver [4], skin [23] and bone marrow [24].

In the present work, we wanted to decipher whether mPER2, one of the central component of the circadian clock machinery, regulates the proliferation and the maturation of NPCs into granule neurons in the hippocampal DG of adult mice. We used the *Per2*^*Brdm1 *^mutant mice line to assess the specific requirement of *mPer2 *in adult neurogenesis. Here, we showed that mPER2 was non-rhythmically expressed in the granule neuronal lineage in the adult DG. In adult *Per2*^*Brdm1 *^mice, we found an increase in both the proliferation of NPCs and the number of immature granule neurons expressing DCX and NeuroD. Strikingly, the pool of adult generated mature granule cells was not enlarged, as one would expect. The accumulation of supernumerary mature granule cells was prevented by apoptosis in the DG of *Per2*^*Brdm1 *^mice. Our results suggest that *mPer2 *belongs to the molecular machinery that underlies the course of adult hippocampal neurogenesis by regulating the early cellular events leading to the production of post-mitotic granule cells in the DG.

## Results

### Constitutive expression of mPER2 in the adult dentate gyrus

**Figure 1:**
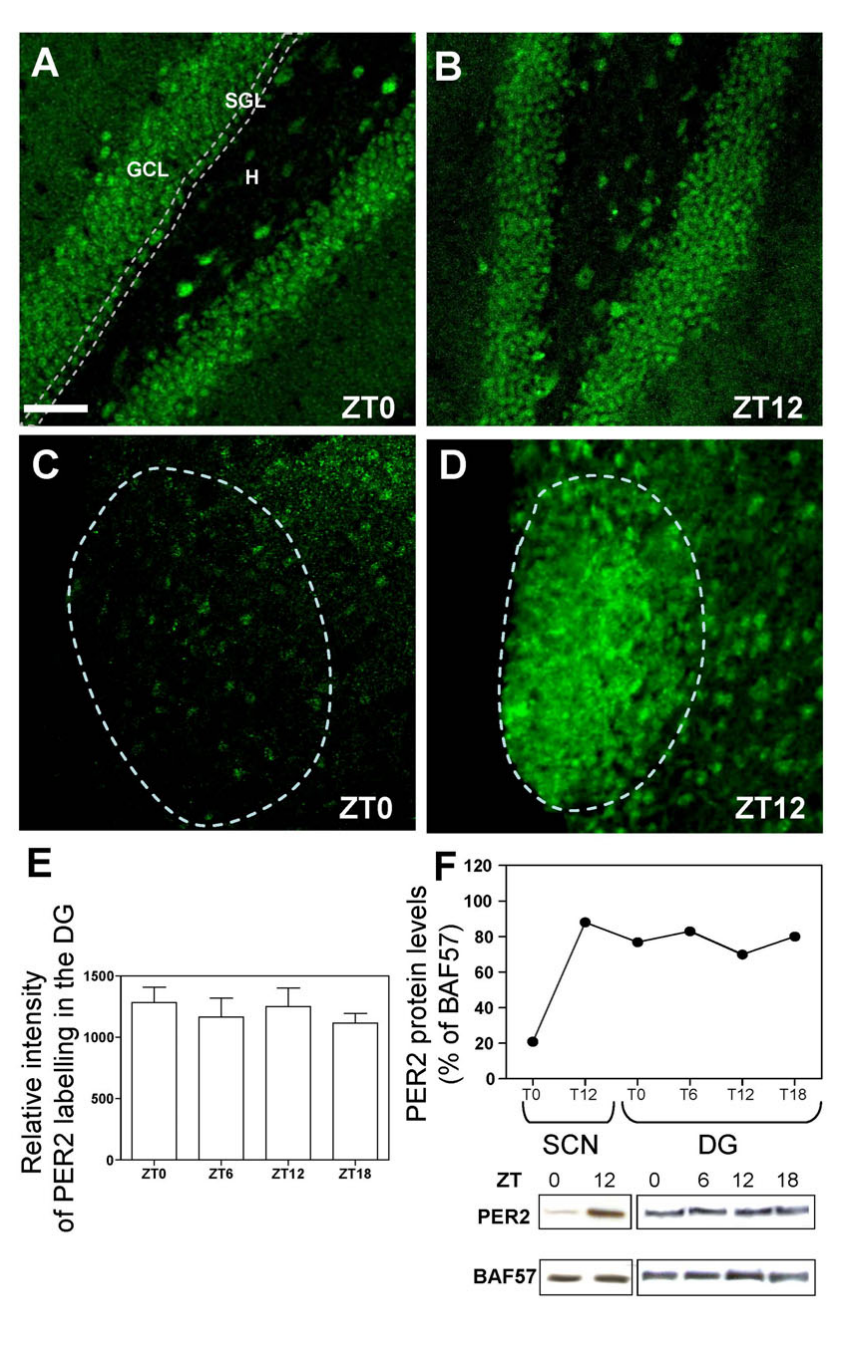
**The mPER2 protein is constitutively expressed in the adult dentate gyrus**. (A-B) Confocal images illustrating the expression of mPER2 in the granule cell layer (GCL), hilar region (H) and subgranular layer (SGL) of the dentate gyrus (DG) from P45 WT mice. No circadian oscillation of mPER2 expression is observed in the DG. (C-D) On the contrary, mPER2 is highly rhythmic in the SCN with a minimum of mPER2 expression at ZT0 (C) and a maximum of mPER2 expression at ZT12 (D). (A-D) Images represent «Z-stack» overlays from 40 μm-thick coronal sections of 5 adjacent confocal planes (step size = 1 μm). (E) The intensity of mPER2 labelling is constant at different circadian time points, i.e. ZT0, ZT6, ZT12 and ZT18 (mean ± SD, n = 3 independent experiments). (F) Levels of PER2 expression were analyzed by Western blot. Analysis were carried out on nuclear protein extracts from micro-dissected SCN and DG (at ZT0, ZT6, ZT12 and ZT18) of P45 WT mice, incubated with mPER2 antibody and BAF57 antibody as internal control. mPER2 antibody yielded a band at the expected size (136 KDa). Results were expressed as percentage of BAF57. Scale bar in A = 50 μm for A-B and 25 μm for C-D.

The expression profile of mPER2 in the DG region of the hippocampus was established by immunohistochemistry using two different polyclonal antibodies on 40 μm-thick coronal sections from P45 adult male mice. Both anti-mPER2 antibodies lead to similar expression patterns (See Additional file 1A–B). mPER2 was highly expressed throughout the adult hippocampus and particularly in the granule cell layer (GCL), subgranular layer (SGL) and the hilus (H) of the DG (Fig. 1A). In order to determine whether mPER2 expression levels could undergo rhythmic oscillations in the DG, immunostainings and Western blots were performed at several circadian time points including ZT0, ZT6, ZT12 and ZT18. Surprisingly, immunolabellings revealed that the level of mPER2 was comparable throughout circadian ZTs (fig. 1E). In contrast, mPER2 expression displayed circadian oscillations in the SCN with a peak of expression at ZT12 (compare fig. 1C and fig. 1D) as previously shown [18,25,26]. Western blots analysis performed on micro-dissected DG confirmed that mPER2 levels were constitutively stable with time, as previously described for its transcript [27], as compared to the SCN, where the maximum of mPER2 expression occurs at ZT12 (Fig. 1F).

### mPER2 is expressed by NPCs and throughout the granule cell lineage in the adult dentate gyrus

**Figure 2:**
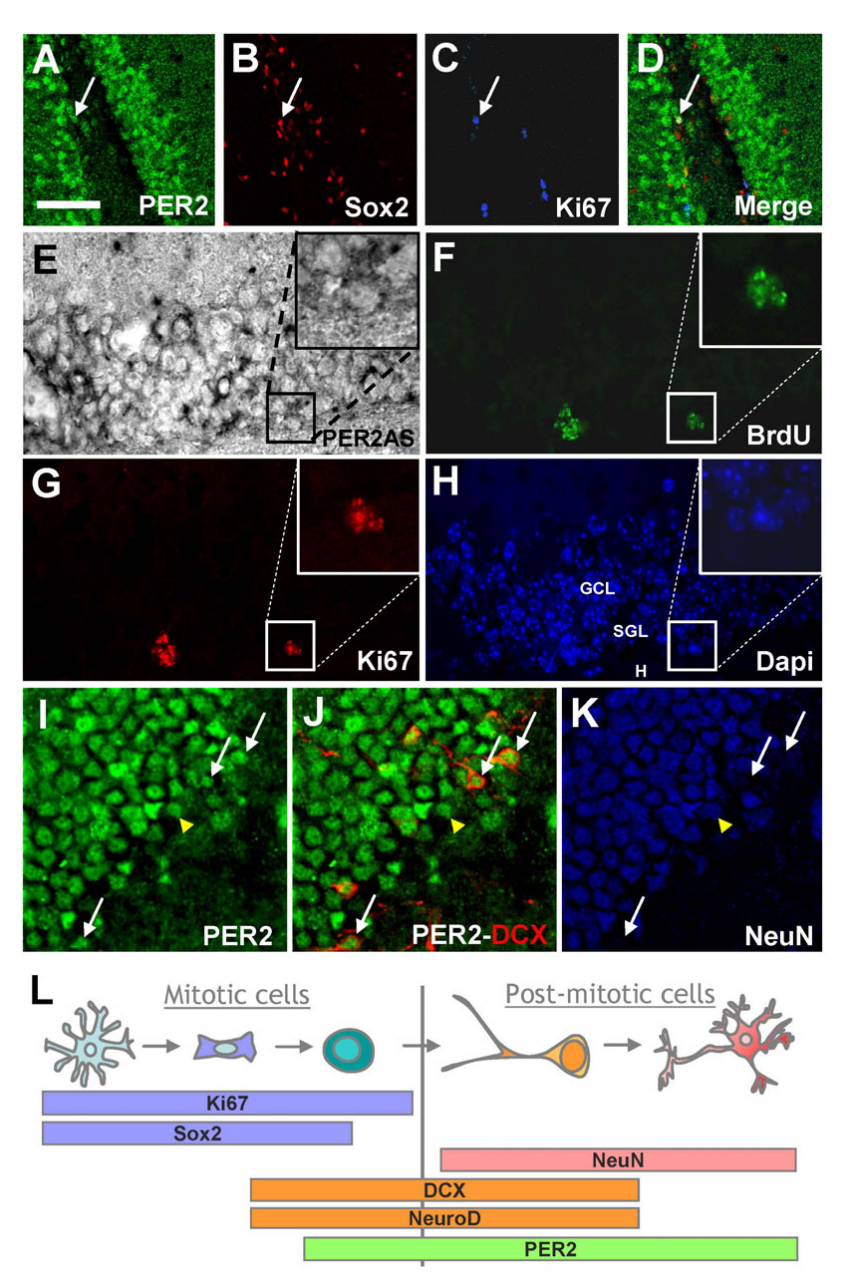
**Phenotype of mPER2-expressing cells in the adult dentate gyrus**. (A-D) Confocal images showing some undifferentiated dividing Sox2^+ ^(B) and Ki67^+ ^(C) cells located in the SGL expressing mPER2 (D). (E-H) *mPer2 *mRNA distribution in the GCL revealed by in situ hybridization (E) and immunostained for BrdU (F) (one i.p. injection at 100 mg/kg of body weight) and Ki67 (G). (H) DAPI was used for nuclear counterstaining. Insets show a mPER2^+ ^cell also labelled for BrdU (F) and Ki67 (G). (I-J) Immature neural precursor DCX^+ ^were found to be mPER2^+ ^(J, white arrows). (I and K) All the mature neurons expressing NeuN were found to be mPER2^+ ^(yellow arrowhead), white arrows point towards mPER2^+^/NeuN^- ^cells. (A-K) All images represent «Z-stack» overlays from 40 μm-thick coronal sections of 5 adjacent confocal planes (step size = 1 μm). (L) Schematic representation of the mPER2 expression in mitotic and post-mitotic cells of the DG. Scale bar in A = 30 μm for A-D and I-K, 50 μm for E-H.

In order to define the phenotypic identity of mPER2^+ ^cells in the adult DG, we performed immunohistochemical stainings with cell type-specific markers. The adult DG contains several cell types including glia-like stem cells (SOX2^+^/S100B^-^/GFAP^+^), transiently amplifying proliferating precursor cells (SOX2^+^/Ki67^+^, some are NeuroD^+ ^and DCX^+^), proliferating neuroblasts (SOX2^-^/DCX^+^/NeuroD^+^/Ki67^+^), immature neurons (DCX^+^/NeuroD^+^/Ki67^-^), mature neurons (NeuN^+^) astrocytes (SOX2^+^/S100B^+^/GFAP^+^) and oligodendrocytes (CNPase^+^) [28,29]. We never found any SOX2^+^/Ki67^-^/mPER2^+ ^cells (data not shown), indicating that glial-like stem cells are mPER2 negative. We observed that some transit amplifying progenitors SOX2^+^/KI67^+ ^expressed mPER2 (Fig. 2A–D). In situ hybridization with antisense *mPer2 *probe, followed by immunohistochemistry, confirmed the expression of *mPer2 *mRNA dividing Ki67^+ ^and BrdU^+ ^cells of the SGL (Fig. 2E–H). Labelling was absent in control sections hybridized with sense probe (data not shown). We quantified dividing cells expressing mPER2 in the adult DG and found that 21,52% ± 13,3% of Ki67^+ ^cells expressed mPER2 (mean ± SD, n = 4). NeuroD and DCX are indeed expressed early in the lineage-determined neuronal cells in the adult hippocampus, i.e. in precursor cells to immature neurons. We observed that some mPER2^+ ^cells expressed DCX and are NeuN negative (Fig. 2I–K), suggesting that mPER2 is expressed in proliferating DCX^+ ^precursor cells. Using double immunostainings to detect mPER2 in immature and mature neurons, we found 79,8 ± 7,6% of NeuroD^+^-, 70,5 ± 4,3% of DCX^+ ^neuroblasts and 100% of NeuN^+ ^neurons (mean ± SD, n = 3) expressed mPER2. Altogether, our findings indicate that in the neuronal lineage, mPER2 expression starts in immature proliferating cells and persists in mature neurons (Fig. 2L).

While mPER2 was not expressed by astrocytes (See Additional file 2A–D) nor oligodendroglial lineage [30] (See Additional file 2E–G), some GABAergic interneurons of the SGL and the hilus were mPER2^+ ^(See Additional file 2H–J).

### The pool of adult hippocampal NPCs is increased in Per2^*Brdm1 *^mice

**Figure 3:**
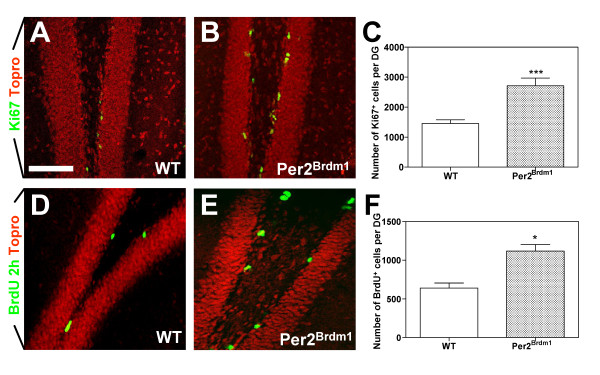
**The mutation of the m*Per2 *gene increases the number of proliferating NPCs in the adult dentategyrus**. (A-B) Images displaying Ki67^+ ^cells (A-B) and BrdU^+ ^cells (D-E) (one i.p. injection at 100 mg/kg of body weight and sacrifice 2 hours later) in the DG from P45 WT (A and D) and *Per2*^*Brdm1 *^(B and E) mice. TO-PRO^®^-3 was used for nuclear counterstaining. All images represent «Z-stack» overlays on 40 μm-thick coronal sections of 5 adjacent confocal planes (step size = 1 μm). (C and F) Histograms representing an unbiased assessment of the number of Ki67^+ ^(C) and BrdU^+ ^(F) cells (mean ± SD, n = 7, Student's *t*-test, ***p < 0,001, *p < 0,05). Scale bar in A = 50 μm for A-B; D-E.

To define whether *mPer2 *controlled the proliferation of hippocampal NPCs, we analyzed the DG of P45 WT and *Per2*^*Brdm1 *^male littermates [27] and observed a significant increase in the total number of Ki67^+ ^cells in the DG of *Per2*^*Brdm1 *^mice as compared to WT (Fig. 3A–C) (mean ± SD, n = 7, ***p < 0,001). Moreover, short-pulsed BrdU incorporation assays (i.e. 2 hours) revealed an increase in S-phase cells in the DG of *Per2*^*Brdm1 *^mice (Fig. 3D–F) (mean ± SD, n = 7, *p < 0,05). Altogether, these results support a role for *mPer2 *in the regulation of the proliferation of NPCs in the adult DG.

### *mPer2 *mutation results in an excess of newly formed neurons in the subgranular layer of the adult dentate gyrus

**Figure 4:**
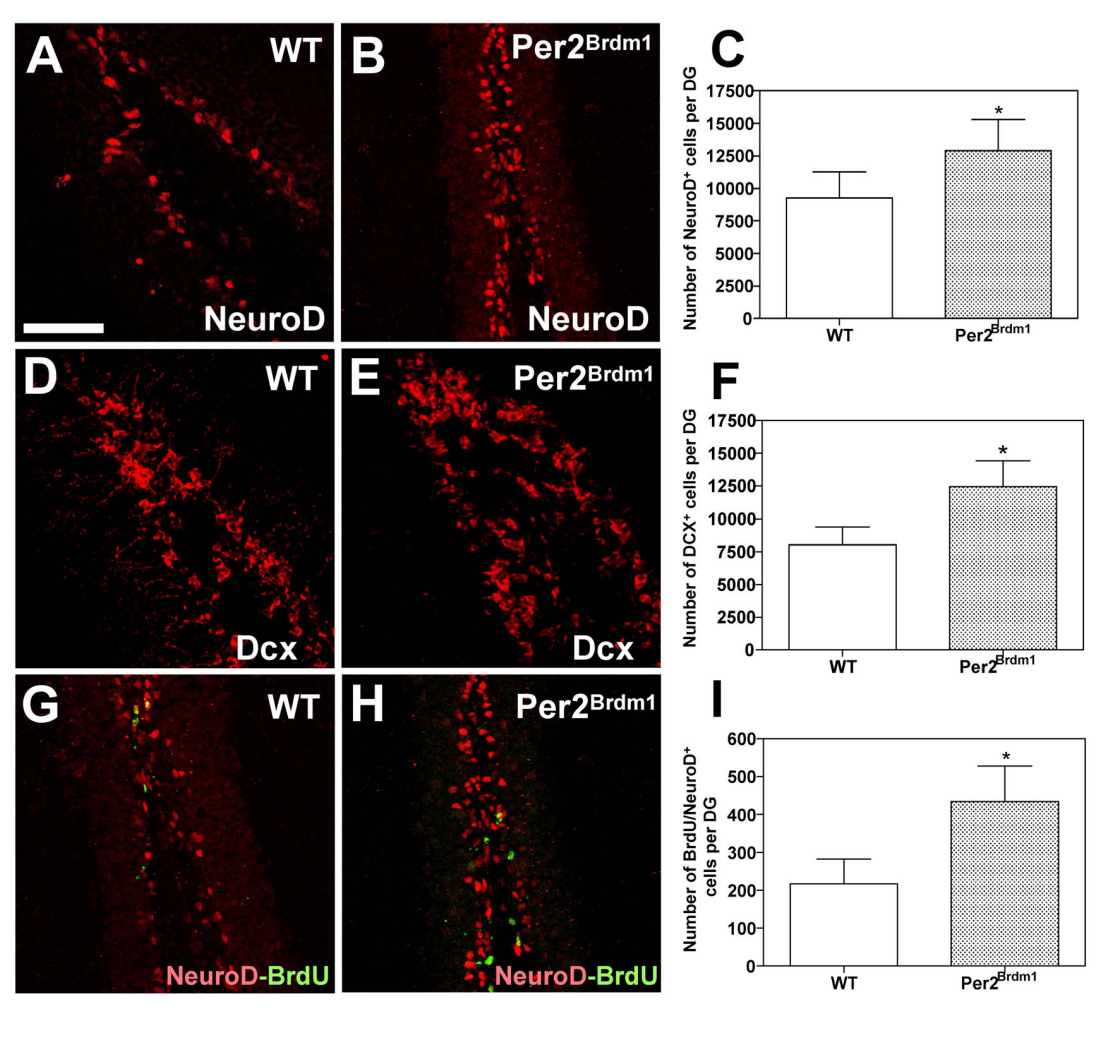
**Increased number of early newborn NeuroD- and DCX-expressing neurons in *Per2*^*Brdm1 *^mutant mice**. DG of P45 WT (A;D;G) and *Per2*^*Brdm1 *^(B;E;H) mice were immunostained for NeuroD (A-B;G-H) and DCX (D-E). Total NeuroD^+ ^(C) and DCX^+ ^(F) cells per section were quantified (mean ± SD, n = 4, Student's *t*-test, *p < 0,05). (G-I) Assessment of the "short-term" neurogenesis. WT and *Per2*^*Brdm1 *^adult mice received a single injection of BrdU (i.p. injection at 100 mg/kg of body weight) and were sacrificed 4 days later. (G-H) DG sections were immunostained with anti-BrdU (green) and anti-NeuroD (red) antibodies. All images represent «Z-stack» overlays on 40 μm-thick coronal sections of 5 adjacent confocal planes (step size = 1 μm). (I) Histograms representing the assessment of the number of immature newborn BrdU^+^/NeuroD^+ ^neurons per DG (mean ± SD, n = 4, Student's *t*-test, *p < 0,05). Scale bar in A = 50 μm for A-H.

In comparison to WT animals, the number of immature neurons expressing NeuroD (Fig. 4A–C) and DCX (Fig. 4D–F) was significantly increased in the SGL of *mPer2 *mutant mice (mean ± SD, n = 4, *p < 0,05). We designed a "short-term" birthdating BrdU incorporation experimental protocol to assess neurogenesis during a short time window of 4 days. In agreement with the expansion of the pool of immature DCX^+ ^and NeuroD^+ ^cells, we observed a 3-fold increase of BrdU^+^/NeuroD^+ ^immature newborn neurons in the DG of *Per2*^*Brdm1 *^mice as compared to WT (Fig. 4G–I)(mean ± SD, n = 4, *p < 0,05).

### *mPer2 *regulates the proliferation and differentiation of NPCs in vitro

**Figure 5:**
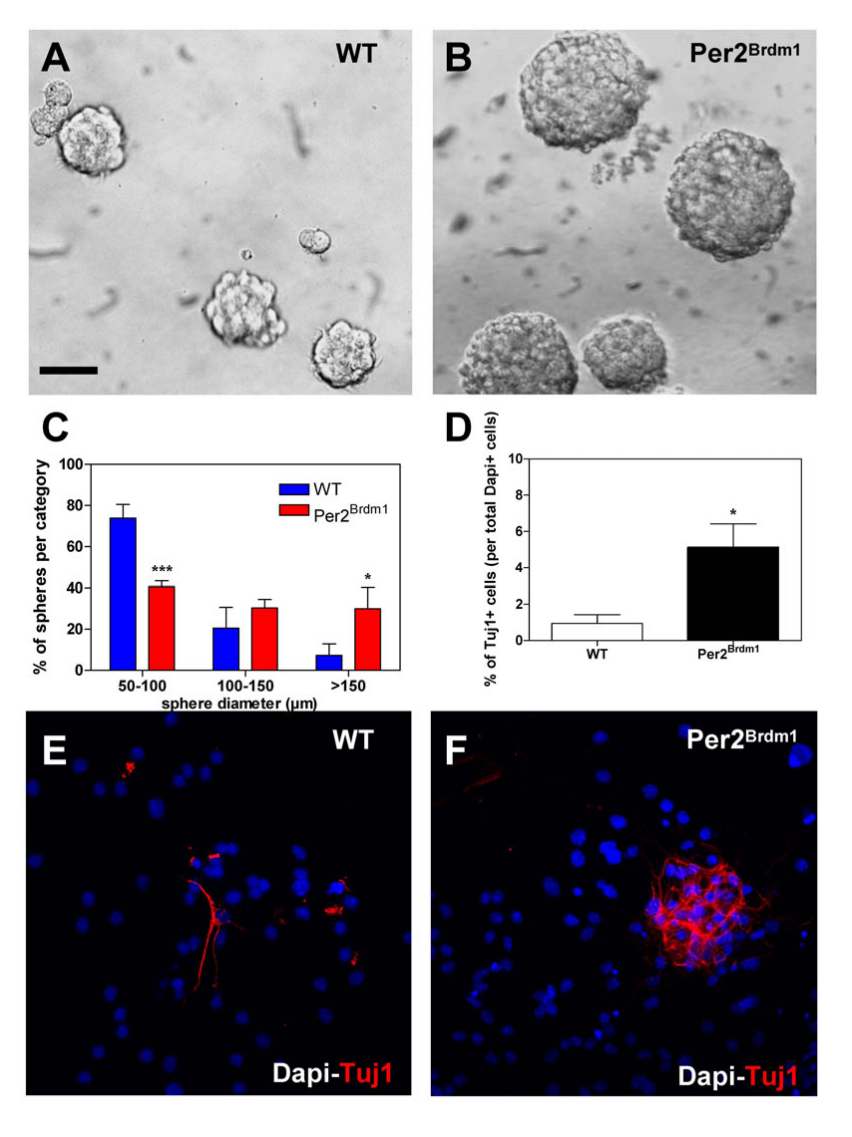
***mPer2 *mutation increased in vitro neural stem/progenitor cell proliferation and differentiation**. (A-C) Representative neurosphere morphologies and size categories for DG of adult P45 WT and *Per2*^*Brm1 *^mice. (C) Spheres were categorized into 3 size groups: (1) 50–100 μm, (2) 100–150 μm and (3)>150 μm in diameter. Data were obtained from three independent experiments and are expressed as mean ± SEM (***p < 0,001 and *p < 0,05 were analyzed by student's *t*-test). (D-F) Differentiating adult NSC cultures for 5 days in vitro on poly-L-ornithin and laminin immunostained for β-III tubulin (Tuj1, red) and DAPI (blue). Increased percentage of neurons was obtained for *Per2*^*Brdm1 *^differentiated neurospheres as compared to WT (D). Results are expressed as mean ± SEM (n = 3 independent experiments, *p < 0,05 analyzed by student's *t*-test). Scale bar in A = 100 μm for A-B and 50 μm for E-F.

To correlate the selective increase of adult NPCs proliferation and neuroblasts differentiation in the DG of *Per2*^*Brdm1 *^mutant mice with a functional in vitro assessment of their proliferative potential ant their multi-lineage differentiation capacity, we compared the potential of WT and *Per2*^*Brdm1 *^NPCs from the DG to form neurospheres and to differentiate into neurons. We showed that *Per2*^*Brdm1 *^NPCs generated significantly larger neurospheres compared with corresponding cells from WT mice (Fig. 5A–B). A careful analysis of the size of secondary neurospheres showed that *Per2*^*Brdm1 *^NPCs generated a reduce number of spheres within the range of 50–100 μm diameter and a significant increase in the number or spheres within >150 μm range (Fig. 5C) (n = 3, mean ± SEM, ***p < 0,001 and *p < 0,05).

To investigate whether the lack of *mPer2 *regulates the differentiation of neurosphere composing cells, we plated neurospheres 5 days in differentiating condition. These cells generate astrocytes, oligodendrocytes (data not shown) and neurons (Fig. 5E and fig. 5F) in both genotypes. Among the progeny of *Per2*^*Brdm1 *^neurospheres, we observed a significant increase of differentiated neurons (Tuj1^+^) (Fig. 5D–F) (mean ± SEM, n = 3, *p < 0,05). Conversely, similar percentages of differentiated astrocytes (GFAP^+^) and oligodendrocytes (O4^+^) were found in both genotypes (data not shown). Altogether, these in vitrodata confirmed our in vivo results and strongly support *mPer2 *as a key player regulating both the proliferation and the neuronal differentiation of NPCs derived from the DG of adult mice.

### Increased apoptotic cell death eliminates supernumerary newborn neurons in the dentate gyrus of adult Per^*2Brdm1 *^mice

**Figure 6:**
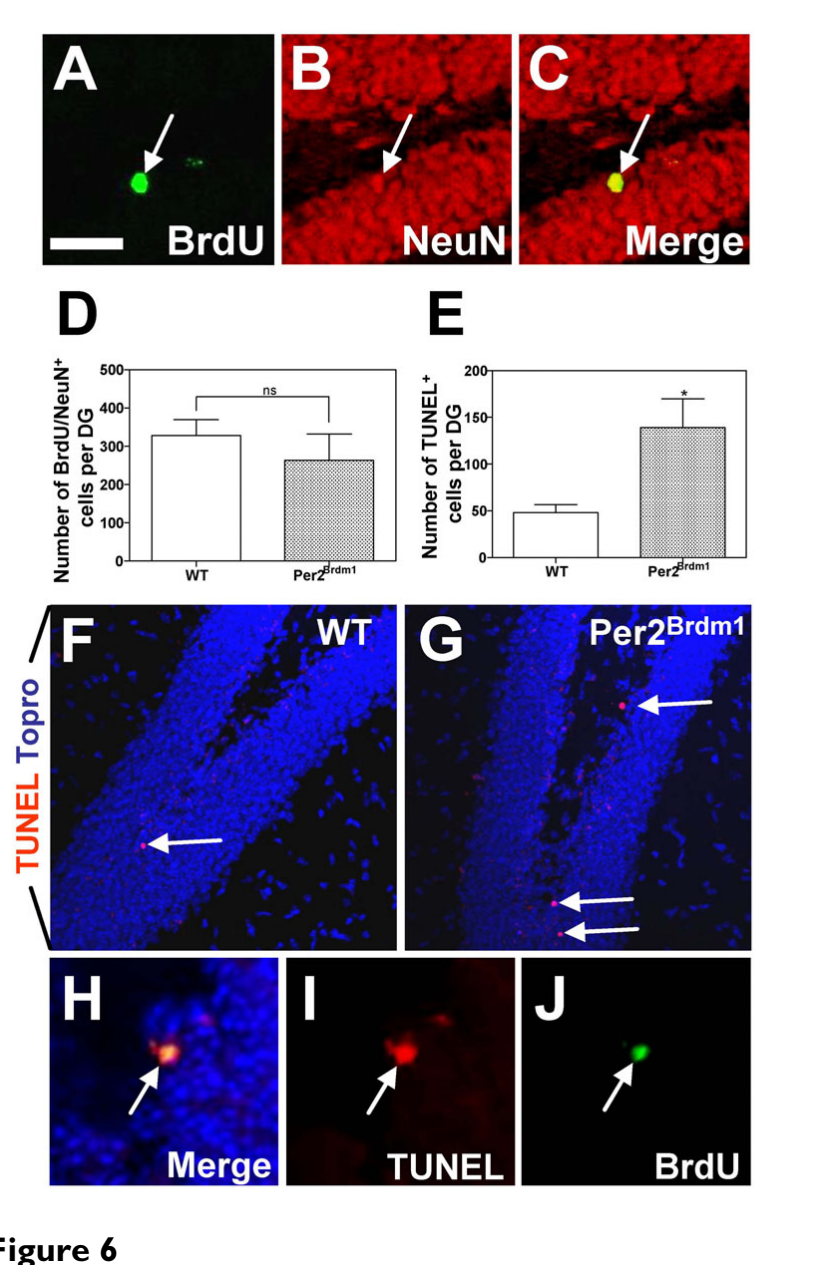
***mPer2 *mutation does not affect end-stage neurogenesis in the adult dentate gyrus because of a compensatory increase in apoptotic cell death**. To assess the "long-term" neurogenesis, WT and *Per2*^*Brdm1 *^mice received a daily injection of BrdU (i.p. injection at 100 mg/kg of body weight) during 5 consecutive days and were sacrificed 3 weeks after the last injection. (A-C) «Z-stack» overlays on 40 μm-thick coronal sections of 5 adjacent confocal planes (step size = 1 μm) displaying the DG of P45 WT mice exposed to the "long-term" BrdU incorporation protocol and stained for BrdU (A), for NeuN (B), or both (C) (white arrow points towards a BrdU^+^/NeuN^+ ^neuron). (D) Histograms representing the overall number of mature newborn BrdU^+^/NeuN^+ ^neurons in the DG of WT and *Per2*^*Brdm1 *^mice (mean ± SD, n = 9 for WT mice and n = 6 for *Per2*^*Brdm1*^, Student's *t*-test, p > 0,05). (F-G) Confocal images displaying TUNEL^+ ^cells in the DG of P45 WT (F) and *Per2*^*Brdm1 *^mutant mice (G). TO-PRO^®^-3 was used for nuclear counterstaining. (E) Total number of TUNEL^+ ^cells (per 40 μm-thick DG section) displays a significant difference between WT and *Per2*^*Brdm1 *^mice (mean ± SD, n = 4, Student's *t*-test, *p < 0,05). (H-J) Newborn cell in the SGL of the DG expressing BrdU, (one i.p. injection of BrdU and sacrifice 4 days later i.e. "short-term" neurogenesis assay) is TUNEL^+^. Scale bar in A = 30 μm for A-C and H-J, 50 μm for E-F, ns = non significant.

We performed "long-term" birthdating BrdU incorporation assays to analyze the final fate and survival of newly formed neurons. Animals were sacrificed 21 days after the end of BrdU injection period. In contrast with the results obtained using a "short-term" BrdU incorporation protocol, we found no difference in the number of mature granule neurons BrdU^+^/NeuN^+ ^in the DG of both WT and *Per2*^*Brdm1 *^mice (Fig. 6A–D) (mean ± SD, n = 9 for WT mice and n = 6 for *Per2*^*Brdm1*^, p = 0,9641).

It is established that during the first 2 to 3 weeks following cell cycle exit, only a fraction of adult-born neurons are functionally integrated into the neuronal network of the DG while most newly formed neurons are naturally dying through apoptosis [20,31]. We thus performed TUNEL stainings to analyze the apoptotic cell death occurring in the DG of *Per2*^*Brdm1 *^and WT male littermates (Fig. 6E–G). The number of TUNEL^+ ^cells was significantly increased in the DG of the *Per2*^*Brdm1 *^mutant mice as compare to WT (Fig. 6E–G) (mean ± SD, n = 4, *p < 0,05). Some TUNEL^+ ^cells were also co-labelled with BrdU (using the "short-term" birthdating assay) indicating that dying cells were newborn neurons (Fig. 6H–J). These results suggest that the newborn NeuroD^+^/DCX^+ ^cells in *Per2*^*Brdm1 *^mice are cleared from the DG through apoptosis (Fig. 6H–J).

## Discussion

The importance of the SCN to generate a multitude of circadian rhythms [32] suggests that most peripheral tissues, including other areas of the brain, are also likely to be under the "circadian" control of the SCN. Our data show that mPER2 clock protein expression is not oscillating in the murine adult DG. This is the first demonstration suggesting that a mammalian clock protein is constitutively expressed in a specific region of the adult brain. Previous findings in agreement with this observation showed a non-circadian expression of *mPer2 *mRNA in the adult DG [18], thymus [33] and testes [34].

It is commonly established that the adult DG is composed of several cell types that include quiescent or slowly dividing and highly proliferating NPCs, immature and mature neurons [29]. Our results indicated that mPER2 expression starts in proliferating NPCs and remains sustained in newborn neurons from the early post-mitotic stage up to mature granule neuronal stage. Similar to the adult DG, murine testes and thymus also retain, to a certain extent, self-renewal capacities during adulthood [34,35]. In these organs, the constant expression of circadian clock gene, including mPER2, strongly suggest that this constitutive expression is a general feature of adult differentiating tissues and that these genes may have new clock-unrelated functions [34].

We wanted here to characterize the putative functions of *mPer2 *in the DG of the hippocampus during adult neurogenesis. Our results indicate that a functional impairment of *mPer2 *(i.e. *Per2*^*Brdm1 *^mice) increased both the number of proliferating NPCs and the production of immature newborn neurons in the DG of adult mice. Hence, the increased number of early newborn post-mitotic neurons that we observed in the DG of *Per2*^*Brdm1 *^mice provided evidence that the expansion in the NPCs pool is not attributable to a delayed cell cycle exit. In line with our in vivo findings, in vitro analyze revealed that cultured NPCs isolated from the DG of *mPer2*^*Brdm1 *^mice generated larger neurospheres as compared to WT. This result argues in favour of *mPer2 *control of NPCs proliferation in neurospheres. In addition, the number of neurons generated from *mPer2*^*Brm1 *^neurospheres significantly increased, suggesting that *mPer2 *regulates the differentiation NPCs into postmitotic neurons. Altogether, these observations obtained from complementary models strongly support a dual function for mPER2 during adult neurogenesis. Our data support the circadian gene *mPer2 *as a new intrinsic regulator of both the proliferation and differentiation in NPCs of the adult murine DG. Interestingly, mPER2 could belong to a global machinery that coordinates the proliferation with the differentiation of NPCs as other molecules (FGF-4, IGF1 and TLR4) controlling both events have recently been identified [36-38]. In line with our observations, the inhibition of mPER2 synthesis in ganglionic eminence cultures results in an increased proliferation [39].

Although the molecular events triggered by mPER2 to regulate these events are not well characterized, the transcription of the proto-oncogene c-myc is deregulated in *Per2*^*Brdm1 *^mice, which consequently are prone to hyperplasia and tumours [40]. Furthermore, the expression of genes involved in cell cycle regulation, such as Cyclin D1, Cyclin A, and p53, is deregulated in many tissues of *Per2*^*Brdm1 *^mice [40,41]. In addition, reduced levels of mPER2 expression were observed in different tumours including lymphoma, breast or endometrial cancer [42-44] and the overexpression of mPER2 in tumour cells led to dramatic growth inhibition and cell cycle arrest [45]. These data are consistent with the observation that *mPer2 *may prevent cell cycle progression and promote the exit of the cell cycle of NPCs in the adult DG. However, the molecular pathways underlying *mPer2 *activity in these cells and which role cell cycle-related genes may play to mediate these effects still need to be elucidated.

By associating with specific cyclins and cyclin-dependent kinases (Cdks) and hence blocking their catalytic activities during cell cycle progression, cyclin-dependent kinase inhibitors promote cell cycle exit at G1 restriction point in various tissues during development [46]. Among them, p21^Cip1 ^and p27^Kip1 ^are expressed in neuroblasts and newly developing neurons in the adult DG [47]. Our preliminary data suggests that p27^Kip1 ^expression broadly overlaps with mPER2 in early post-mitotic hippocampal immature neurons (data not shown). In addition, the proliferation and the number of DCX^+ ^cells in the adult DG, are increased in p21^cip1-/- ^mice [48,49]. Interestingly, it has been shown recently that the circadian clock component BMAL1, regulates the expression of p21^cip1 ^in hepatocytes [50]. Taken together, these observations suggest p21^cip1 ^and p27^kip1 ^as strong candidates that could act downstream *mPer2 *to inhibit NPCs proliferation in the adult DG.

Unexpectedly, the number of newly formed mature granule neurons was similar in WT and *Per2*^*brdm1 *^mice. During the first weeks following cell cycle exit, only a part of the newly generated cells find their definitive place in the hippocampal circuitry while around 50% of them are naturally eliminated through apoptosis [17,20,31]. Previous works have shown that programmed cell death is a physiological process that occurs during neurogenesis in the adult DG [51]. In *Per2*^*brdm1 *^mice, despite an increased proliferation of NPCs and an increased number of early newborn neurons, we did not find any significant enlargement of the DG. However, we observed more TUNEL^+ ^cells in the DG of *Per2*^*brdm1*^. We can suggest two possible explanations for this increased cell death phenomenon. First, mPER2 may regulate the expression of pro-survival genes that would be underexpressed in *Per2*^*brdm1 *^and lead to the death of newly born granule cells in the DG. Such selective or combined effects on distinct phases of neurogenesis have already been reported. For instance, an enriched environment is known to affect only cell survival [14,52] whereas physical exercise [12], seizures [53] or absence of TLR4 [38] increase both cell proliferation and cell survival. Alternatively, the excessive cell death observed in *Per2*^*brdm1 *^mouse DG could be an indirect consequence, i.e. a secondary and compensatory response to increased cell proliferation in *Per2*^*brmd1 *^mice. In this case, the production of new neurons in the hippocampus would be regulated by a physiological and homeostatic parapet. Similar homeostatic regulations of the formation of new neurons have been described for mCD24-deficient mice [54] or after a treatment with antidepressant drugs [55]. Reciprocally, enhanced survival of new granule cells was reported after hampering cell proliferation with methylazoxymethanol or in synapsin III knock-out mice [56,57]. Excess number of immature, DCX^+ ^and NeuroD^+ ^neuroblasts as reported in the DG of *Per2*^*Brdm1 *^mice may increase competition for trophic signals required for the maintenance of a mature DG neuronal phenotype as suggested previously [58].

Altogether, these data implied that precursor cell proliferation, cell survival, and cell differentiation are all essential events that regulate the final number of new granule neurons in the adult hippocampus. Importantly, our results show that an increased neuronal cell death prevents the accumulation of supernumerary newborn neurons in *Per2*^*Brdm1 *^mutant mice.

## Conclusion

The present work has unravelled an original functional link between the constitutive expression of the circadian gene *mPer2 *and the intrinsic control of NPCs proliferation, cell death and neurogenesis in the adult DG. These findings now raise new questions that include the identification of the presumably complex molecular network activated by *mPer2 *and required to tune the fine balance existing between cell proliferation, cell death and differentiation during adult hippocampal neurogenesis. To what extent the expression of circadian genes in the DG of the hippocampus is related or synchronized by central SCN induced rhythms remains to be elucidated. This work also emphasizes that a better understanding of the molecular cues that control circadian gene expression in the hippocampus may open new avenues to regulate neurogenesis and its yet unknown physiological function in the adult hippocampus.

## Methods

### Experimental animals

The *mPer2*^*Brdm1 *^mutant mice were from Zheng et al. [27] and the CNP-EGFP mice were from Yuan et al. [30], and were propagated in the animal facility of the university of Liège. The *mPer2 *gene is coding for a transcription factor possessing a PAS (Period/Arnt/Single minded) dimerization domain that is deleted in the *Per2*^*Brdm1 *^mutant mice. The phenotype of our deletion mutation is consistent with a loss-of-function mutation [27]. *Per2*^*Brdm1 *^mice maintained in a 12 hours light/12 hours night cycle present a normal locomotor activity as compare to WT mice [27]. We studied adult 45 days-old (P45) males WT and *Per2*^*Brdm1 *^mutant littermates produced from heterozygous crosses. We also used P45 CNP-EGFP mice. Artificial light was provided daily from 7 AM (Zeitgeber time ZT0) to 7 PM (ZT12) (12 hours of light/12 hours of darkness with room temperature (RT) and humidity kept constant). All animals were used in accordance with the declaration of Helsinki and following the guidelines of the Belgian ministry of agriculture in agreement with EC laboratory animal care and use regulation (86/609/CEE, CE of J n°L358, 18 December 1986).

### Processing of tissue sections

Mice were anaesthetized with Nembutal (CEVA Santé Animal, Brussels, Belgium) at selected Zeitgeber times (ZT0, ZT6, ZT12 and ZT18) before being subjected to intracardiac perfusion with 4% paraformaldehyde in PBS. Brains were removed, cryoprotected for 48 hours in 30% sucrose/PBS solution and cut with a cryostat into 12 μm-thick-coronal sections for in situ hybridization or into 40 μm free-floating coronal sections for immunohistochemistry.

### Immunohistochemistry

Free-floating sections were blocked for 30 minutes with blocking solution [PBS containing 5% normal donkey serum (Jackson Immunoresearch Laboratories, West Grove, PA, U.S.A.) and 0,3% triton X-100 (Sigma-Aldricht, Bornem, Belgium)]. They were then incubated one night at 4°C in blocking solution with primary antibodies directed against mPER2 (1:250, rabbit polyclonal IgG, at, Alpha Diagnostic international, ADi, San Antonio, TX, U.S.A., and rabbit polyclonal IgG, 1:250, Santa Cruz, SC-25363, Heidelberg, Germany). The two antibodies give rise to similar immunolabellings. We used also primary antibodies directed against NeuN (1:250, Millipore, Brussels, Belgium), doublecortin (DCX, 1:500, Santa Cruz), NeuroD (1:100, Santa Cruz), GABA (1:750, Millipore), Sox2 (1:250, Santa Cruz) and S100β (1:500, DAKO, Glostrup, Denmark). After washing three times with PBS, sections were reacted at RT for 2 hours with RRX-, FITC- or Cy 5-conjugated secondary antibodies (1:500, Jackson Immunoresearch Laboratories). After washing, sections were finally incubated with the nucleic acid stain TO-PRO^®^-3 (1:1500 in PBS, Molecular Probes, Eugene, U.S.A.) and coverslipped using the Vectashield mounting medium (Hard Set Mounting Medium, Vector laboratories, Burlingame, U.S.A.), or directly mounted with the Vectashield mounting medium containing the nucleic acid counterstain DAPI (Vector laboratories). The slides were stored in the dark at 4°C. Controls were performed by omitting primary antibodies, which in all cases resulted in a complete loss of immunofluorescence signal.

### Demonstration of antibody specificity

The synthetic blocking peptide PER21-P is incubated with mPER2 antibody. PER21-P (approximately 5 μg) and control PBS solutions were incubated overnight at 4°C both in the presence of anti-mPER2 antibody (approximately 1 μg) diluted at usual concentration (1/250). After centrifugation at 15000 RPM to pellet any immune complexes, the supernatants were removed and used for immunostainings (See Additional file 1).

### Assessment of cell proliferation and neurogenesis

Brain sections were stained for Ki67 (1:100, mouse, BD Biosciences, San Jose, CA, U.S.A.), a nuclear marker of cell division that is expressed in all phases of the cell cycle (G1-S-G2-M). In order to study specifically S-phase dividing cells, WT and *Per2*^*Brdm1 *^mice were injected with 5'-bromo-2-deoxyuridine (BrdU) (i.p. injection at 100 mg/kg of body weight; Sigma-Aldrich) and were sacrificed 2 hours later. For immunohistochemical detection of BrdU-incorporating nuclei, free-floating sections were incubated in 2 N HCl for 30 minutes at 37°C. Sections were then incubated in a 0,1 M borate buffer solution (pH 8.5), rinsed three times in PBS and stained for BrdU (1:500, rat IgG2a, Immunosource, Raleigh, U.S.A.) as described above.

To assess the total number of newborn neurons in the adult DG, P45 WT and *Per2*^*Brdm1 *^mice received one injection of BrdU (i.p. injection at 100 mg/kg of body weight) and were sacrificed four days later ("short-term" assessment of neurogenesis) or received one daily injection during five days and sacrificed three weeks later ("long-term" assessment of neurogenesis). We then analyzed the fate of BrdU-tagged newborn cells in the adult DG by coupling BrdU and NeuN immunostainings.

### Cell death detection

To study the cell death, the terminal dUTP-biotin nick end labelling (TUNEL) was used [59] on 40 μm-thick free-floating sections of P45 WT and *Per2*^*Brdm1 *^mice. Sections were treated 30 minutes with 1% triton X-100 and pre-incubated 10 minutes in TdT buffer (30 mM Tris, pH 7,5, containing 140 mM sodium cacodylate and 1 mM cobalt chloride). Sections were then incubated in TdT buffer containing 300 U/ml TdT (Roche Applied Science) and 6 μM biotinylated dUTP (Roche Applied Science) in a humid chamber for 90 minutes at 37°C. The reaction was stopped by a 15 minutes rinse at RT in TB buffer (300 mM NaCl, 30 mM sodium citrate, pH 7,4). Sections were incubated 10 minutes with 2% BSA (bovine serum albumin fraction V, Sigma-Aldrich) in PBS to minimize non-specific staining. Biotin-dUTP-labeled sections were then incubated with Alexa568-conjugated streptavidin (1:1000, Jackson Immunoresearch Laboratories) for one hour at RT. Finally, fluorescent immunohistochemical staining for BrdU was performed on TUNEL labelled sections as described above.

### In situ hybridization

The sequence of the antisense probe was made from a DNA template corresponding to *mPer2 *nucleotides 229–768 (AF036893). RNA probes were prepared by in vitro transcription with the digoxigenin labelling kit (Roche Applied Science) using T7 RNA polymerase for sense probe and T3 polymerase for antisense. 12 μm-thick adherent sections were acetylated 15 minutes, and dehydrated. After three washes with PBS-Tween 0,1%, sections were pre-hybridized with pre-warmed hybridization buffer (Amresco, Ohio, U.S.A.) for 60 minutes at 55°C. Sections were then hybridized one night at 55°C with 800 ng/ml *mPer2 *RNA probe. The next day, sections were washed twice with pre-warmed washing buffer (formamide 50%, 0,1% of tween, SSC 20× in H_2_0) for 60 minutes at 65°C. Sections were then incubated one night at 4°C with an anti-digoxigenin antibody coupled to alkaline phosphatase (1:2000, Roche Applied Science) in buffer composed of Tris-HCl 100 mM pH 7,5, NaCl 150 mM, 0,1% of Tween-20, and 10% goat serum (DAKO). Sections were overlaid with 200 μl filtered NBT/BCIP/Tween-20 0,1% solution (Sigma-Aldrich) between coverslips for six hours. Sections were then washed three times in PBS to stop the reaction and post-fixed 15 minutes with PFA 4%. Sections were then processed for immunohistochemistry as described above.

### Neural stem/progenitor cells culture

The primary cell culture technique used was the neurosphere assay [60]. The DG of three WT or three *Per2*^*Brdm1 *^P10 mice were removed and incubated whit Earl's Balanced Salt Solution (Invitrogen, Merelbeke, Belgium) containing one mg/ml of papain (Worthington, Lakewood, U.S.A.) and 500 μl of DNAse 0,1% (Sigma-Aldrich) for 35 minutes at 37°C. After mechanical dissociation, cells were suspended in culture (10^4 ^cells/cm^2^) in DMEM/F12/B27 medium with epidermal growth factor (20 ng/ml, PeproTech, London U.K.) and basic fibroblast growth factor (10 ng/ml, PeproTech). Primary spheres obtained after five days were dissociated in enzyme solution (as above) and dissociated cells were re-suspended into fresh medium solution at 10^4 ^cells/cm^2^. It has been previously demonstrated that culturing cells at this density resulted in clonal neurosphere colonies, as formed in single-cell cultures. Neurospheres do not arise as a result of cell aggregation at this cell culture density [61,62]. All experiments were performed between passage two and three. Neurospheres size was determined by measuring the diameter of individual neurosphere (assuming a spherical shape) using ImageJ software.

Neural stem cell differentiation was induced in neurospheres plated onto poly-L-ornithin (15 μg/ml) and laminin (10 μg/ml) coated coverslips in DMEM/F12 supplemented with 1% fetal bovine serum (Invitrogen), B27 with vitamin A (Invitrogen) and 20 ng/ml of brain-derived neurotrophic factor (PeproTech). After five days of differentiation, neurospheres were fixed in 4% PFA and processed for immunocytochemistry as described above with primary antibodies against GFAP (1:100, DAKO), β-III tubulin (1:500, Tuj1 clone, Babco, Richmond, CA, U.S.A.), O4 (1:100, Millipore) and nestin (1:250, Novus, Littleton, U.S.A.). All these experiments were done in triplicate.

### Image acquisition and analysis

Immunostained sections were imaged using the Olympus Fluoview FV1000 confocal system equipped with the Olympus IX81 inverted microscope (Olympus Europa GmbH, Hamburg, Germany). For each 40 μm-thick section, the whole DG, was Z-scanned using the 40× objective, and the composite of the «Z-stack» images was analyzed. All sections prepared for comparison were stained at the same time, in the same conditions and were selected at the same stereological location.

Drawing a box across the GCL and measuring the mean intensity value in pixels determined fluorescence intensity of mPER2 levels. Background value taken from unlabeled areas was subtracted from measured value for every field analyzed. Three separate sections were analyzed, in three different animals and Prism software was used for statistical analysis, and Student's *t*-test was used to determine significance levels.

To estimate the total number of immunolabelled cells in the GCL of the DG, we used a modified version of the optical fractionator method, as recently described [63,64]. Briefly, a systematic random sampling of every sixth 40-μm coronal section (240 μm) along the rostro-caudal axis of the DG (-1.06 mm to -3.80 mm form bregma) was selected from each animal and processed for immunohistochemistry. Immunopositive cells in the GCL were counted under a 40×-magnification microscope objective. The total number of positive cells was estimated by multiplying the results by six (because every sixth section were used) to provide an estimate of the total number of cells per DG.

To estimate any volumetric discrepancy between the DG of WT and *Per2*^*Brdm1 *^mice, we compared similar GCL areas between WT and *Per2*^*Brdm1 *^mice using adjacent coronal sections throughout the entire rostral/caudal extent of the hippocampus (from Bregma -1.06 mm to Bregma -3.80 mm) as previously described [65]. Every sixth 40 μm-thick section has been NeuN immunostained and imaged. Using ImageJ software, the GCL has been outlined and the area obtained. Volume has been estimated using the formula ΣAxTxI, where ΣA is the sum of the area measured on each section, T is the section thickness, and I is the number of section intervals. The number of sections varied between genotypes; thus, sampling began at the first indication of a complete DG and ended when the DG was no longer visible. We showed that there were no volumetric differences between GCL of the hippocampus of the WT and *Per2*^*Brdm1 *^mice (See Additional file 3).

### Western Blotting

Mice were killed at ZT0, ZT6, ZT12 or ZT18 by cervical dislocation. Brains from P45 WT mice were removed, placed rostral-side up in 35 mm Petri dishes and covered with a 3% solution of low-gelling temperature agarose at 37°C and then immediately placed at 4°C to harden the agarose solution. Brains were cut on a vibratome (Leica, Zaventem, Belgium) into viable 700 μm coronal sections in ice-cold PBS solution containing 30% glucose, and the DG of the hippocampus or the SCN were removed under a dissecting microscope. Tissues were lysed on ice using 200 μl of lysis buffer [Hepes 10 mM pH 7,9, KCl 10 mM, MgCl_2 _2 mM, EDTA 0,1 mM, Igepal™ 0,1% (Sigma-Aldrich), DTT 1 mM, PMSF 1 mM and protease inhibitors cocktail (Roche Applied Science)]. After five minutes on ice, the samples were centrifuged 5 minutes at 2000 g. The pellet was washed two times with 500 μl of washing buffer [Hepes 10 mM, pH 7,9, KCl 20 mM, MgCl_2 _2 mM, EDTA 0,1 mM, DTT 1 mM, PMSF 1 mM, and protease inhibitors cocktail]. The samples were then homogenized with 50 μl of extraction buffer [Hepes 20 mM pH 7,9, MgCl2 1,5 mM, EDTA 0,02 mM, NaCl 0,63 mM, H_2_0, Glycerol 25%, DTT 0,5 mM, PMSF 1 mM, protease inhibitors cocktail] and incubated for 30 minutes on ice and 10 minutes at -80°C. The homogenates were then centrifuged at 14000 g for 30 minutes at 4°C. The supernatants were used as nuclear fraction and the protein concentration was determined using RC DC Protein Assay (Bio-Rad, Nazareth, Belgium). Nuclear protein lysates (20 μg) were then mixed with an equal volume of loading buffer (glycerol 20%, SDS 4%, Tris 100 mM, β-mercaptoethanol 5% and bromophenol blue) before being boiled for three minutes. Proteins were then loaded on a 7,5% SDS-polyacrylamide gel electrophoresis, and the gel run 20 minutes at 100 volts. Proteins contained in the gel were transferred on a polyvinylidene difluoride membrane (Amersham, Roosendaal, The Netherlands) by semi-dry electroblotting in transfer buffer (Glycine 192 mM, Tris 25 mM and methanol 20%). Membranes were then blocked one hour at RT in 0,2% I-BLOCK (TROPIX, Bedford, MA, U.S.A.) diluted in TBS supplemented with 0,05% Tween 20 (Bio-Rad) (TTBS) corresponding to the blocking buffer. Membranes were incubated one night at 4°C in the blocking buffer containing the primary antibody directed against mPER2 (H-90) (1:2500 in the blocking buffer, SC-25363, Santa Cruz or Per21 A, ADi). After washing three times with TTBS, the membranes were incubated with peroxidase-conjugated polyclonal anti-rabbit antibody (1:3000, ABCAM, Cambridge, U.K.) one hour at RT. Blots were then washed extensively with TTBS and developed using enhanced chemoluminescence ECL substrate (Pierce, Aalst, Belgium). Membranes were stripped with Restore™ blot stripping buffer (Pierce), wash three times in TTBS and incubated one night at four °C with BAF57 antibody (1:2500, rabbit, Sigma-Aldrich). After washing, the membranes were incubated with peroxidase-conjugated anti-rabbit antibody (1:3000, ABCAM) and developed as described above. Quantification of the bands was carried out by densitometric analysis using Image J software.

## Abbreviations

BrdU: 5'-bromo-2-deoxyuridine; cdk: cylin-dependent kinase; DCX: doublecortin; DG: dentate gyrus; DNA: deoxyribonucleic acid; GCL: granule cell layer; H: hilus; i.p: intra-peritoneal; *mPer1*: period 1 gene; *mPer2*: period 2 gene; mPER2: period 2 protein; *mPer3*: period 3 gene; mRNA: ribonucleic acid; NPC: neural progenitor/stem cell; PBS: phosphate buffer saline; *Per2*^*Brmd1*^: per2 mutant mice; PFA: paraformaldehyde; RT: room temperature; SCN: suprachiasmatic nuclei; SGL: subgranular layer; TBS: tris buffer solution; TTBS: tween-20/tris buffer solution; TUNEL: terminal dUTP-biotin nick end labelling; WT: wild-type; ZT: Zeitgeber.

## Authors' contributions

LB, PB, RV carried out the studies and participated in the data analysis. LB, LN, SB, BM conceived the study, participated in its design and helped to draft the manuscript. GM and PM contributed to the conception and design of the project. UA produced the mice and helped in the last version of the manuscript. All the authors read and approved the final manuscript.

## Supplementary Material

Additional file 1 **Expression of mPER2 protein in adult dentate gyrus sections using different antibodies**. (A-B) Panels representing confocal images of DG sections simultaneously processed for immunostaining with ADi *Per2 *antibody (A) or Santacruz *Per2 *antibody (B) at ZT12. The two antibodies recognize identically mPER2. (C) Western blot analysis were carried out on nuclear protein extracts from micro-dissected SCN and DG at ZT12. Both ADi and Santacruz antibodies yielded bands at the expected size (136 kDa). (D-I) anti-PER2 antibody was pre-incubated with Per21-P blocking peptide (D-F) or not (G-I) before immunolabelling procedure. Nuclear counterstaining was obtained using To-Pro^®^3 (E, H). Scale bar in A = 50 μm for A-B, 40 μm for D-I. Click here for file

Additional file 2 **mPER2 is expressed in GABAergic neurons but not in astroglial and oligodendroglial cells in the adult dentate gyrus**. White arrow point towards a neural stem/progenitor cell located in the SGL of the DG expressing mPER2 (A) and Sox2 (B). (C) This cell is negative for the astroglial marker S100β (C). Astroglial cell co-expressing S100β and Sox2 (white arrowhead A-D) is mPer2 negative. (E-G) mPER2 and CNP-EGFP expression in the DG of P45 WT mice. White arrows point towards CNP-EGFP^+ ^oligodendrocytes that were all mPER2^-^. (H-J) A significant proportion of mPER2^+ ^neurons (white arrows) in the hilar region (H) and granule cell layer (GCL) of the DG were GABA^+^. Scale bar in A = 25 μm for A-D and 50 μm for E-J. Click here for file

Additional file 3 **No significant difference between the volume of the dentate gyrus in WT and *Per2*^*Brdm1 *^mice**. (A-B) Confocal images of coronal sections of the DG counterstained with NeuN in order to study the absence of morpho-volumetric differences between WT and *mPer2*^*Brdm1 *^mutant mice at the same level of the rostro-caudal axis. (C) We have compared similar hippocampal areas between WT and *mPer2*^*Brdm1 *^mutant mice and observed that the volume of the granule cell layer (GCL) was not significantly different between the two genotypes (Student's *t*-test, n = 3, p > 0,05). Scale bar in A = 300 μm for A-B. Click here for file
