# Supplementary figures and images for: Period 2 regulates neural stem/progenitor cell proliferation in the adult hippocampus
Source: BMC Neurosci. 2009 Mar 27;10:30. doi: 10.1186/1471-2202-10-30 (PMC2714160; doi:10.1186/1471-2202-10-30)

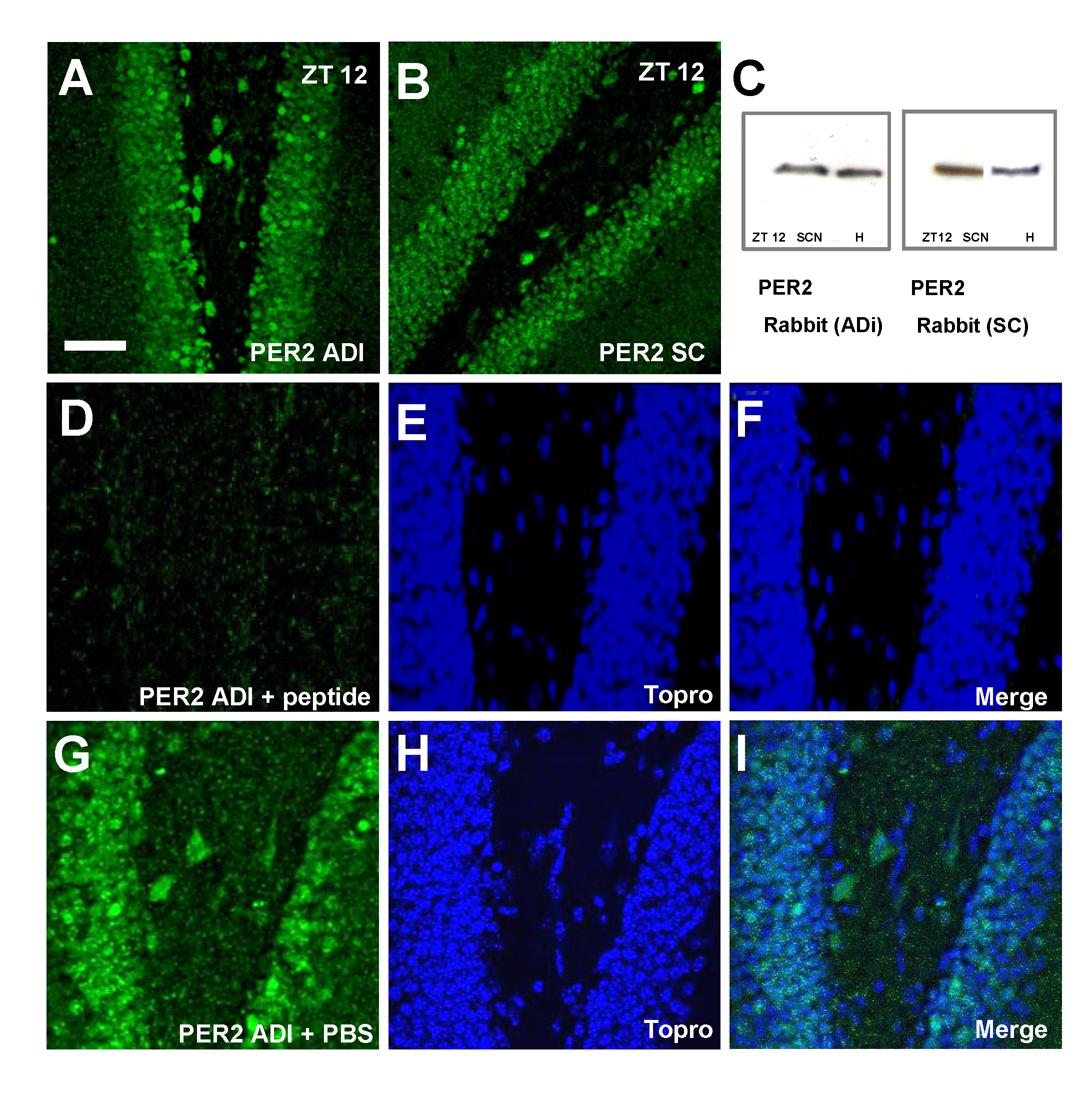

Supplement: Additional file 1 — Expression of mPER2 protein in adult dentate gyrus sections using different antibodies. (A-B) Panels representing confocal images of DG sections simultaneously processed for immunostaining with ADi Per2 antibody (A) or Santacruz Per2 antibody (B) at ZT12. The two antibodies recognize identically mPER2. (C) Western blot analysis were carried out on nuclear protein extracts from micro-dissected SCN and DG at ZT12. Both ADi and Santacruz antibodies yielded bands at the expected size (136 kDa). (D-I) anti-PER2 antibody was pre-incubated with Per21-P blocking peptide (D-F) or not (G-I) before immunolabelling procedure. Nuclear counterstaining was obtained using To-Pro®3 (E, H). Scale bar in A = 50 μm for A-B, 40 μm for D-I. [file 1471-2202-10-30-S1.tiff]

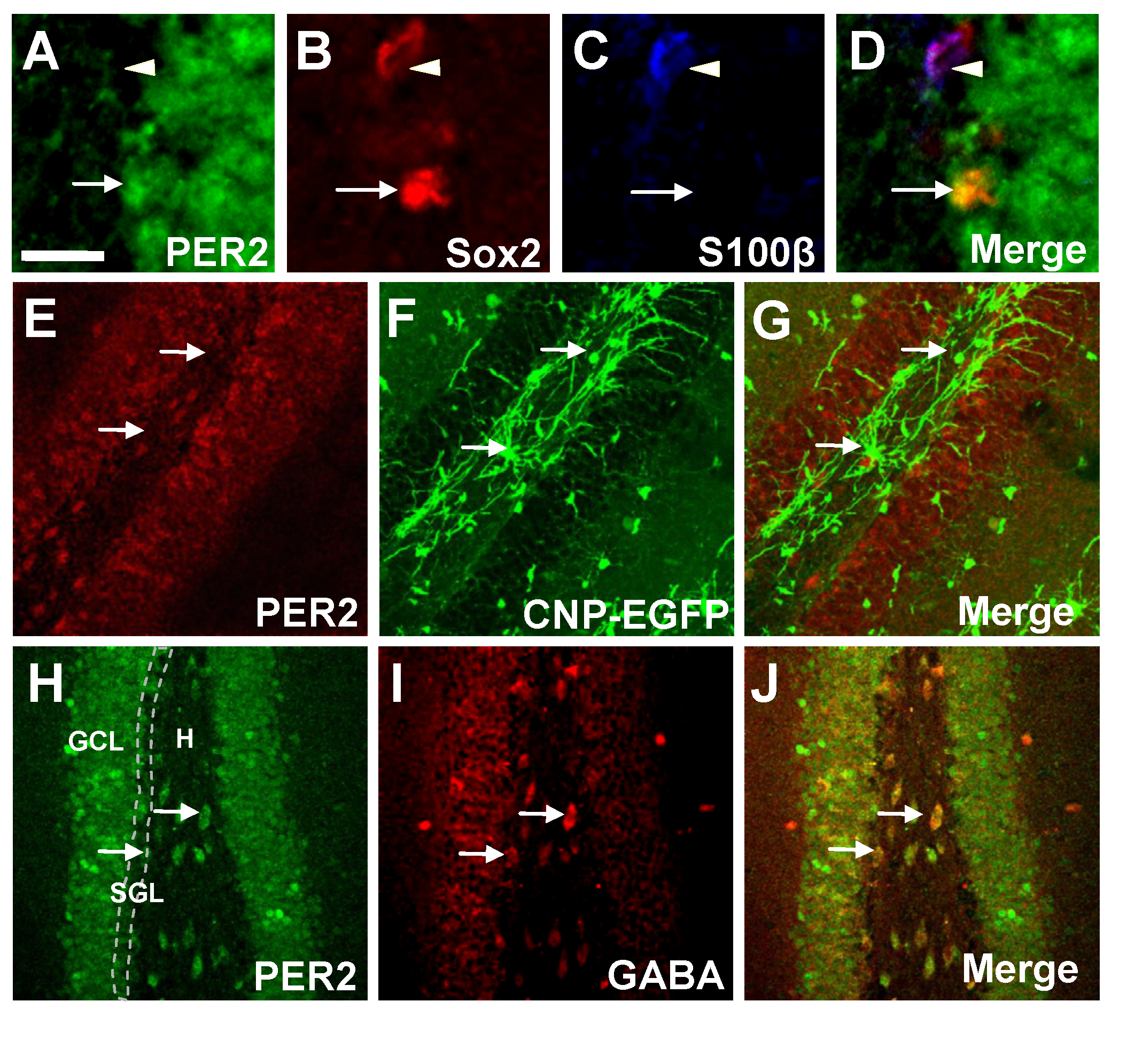

Supplement: Additional file 2 — mPER2 is expressed in GABAergic neurons but not in astroglial and oligodendroglial cells in the adult dentate gyrus. White arrow point towards a neural stem/progenitor cell located in the SGL of the DG expressing mPER2 (A) and Sox2 (B). (C) This cell is negative for the astroglial marker S100β (C). Astroglial cell co-expressing S100β and Sox2 (white arrowhead A-D) is mPer2 negative. (E-G) mPER2 and CNP-EGFP expression in the DG of P45 WT mice. White arrows point towards CNP-EGFP+ oligodendrocytes that were all mPER2-. (H-J) A significant proportion of mPER2+ neurons (white arrows) in the hilar region (H) and granule cell layer (GCL) of the DG were GABA+. Scale bar in A = 25 μm for A-D and 50 μm for E-J. [file 1471-2202-10-30-S2.tiff]

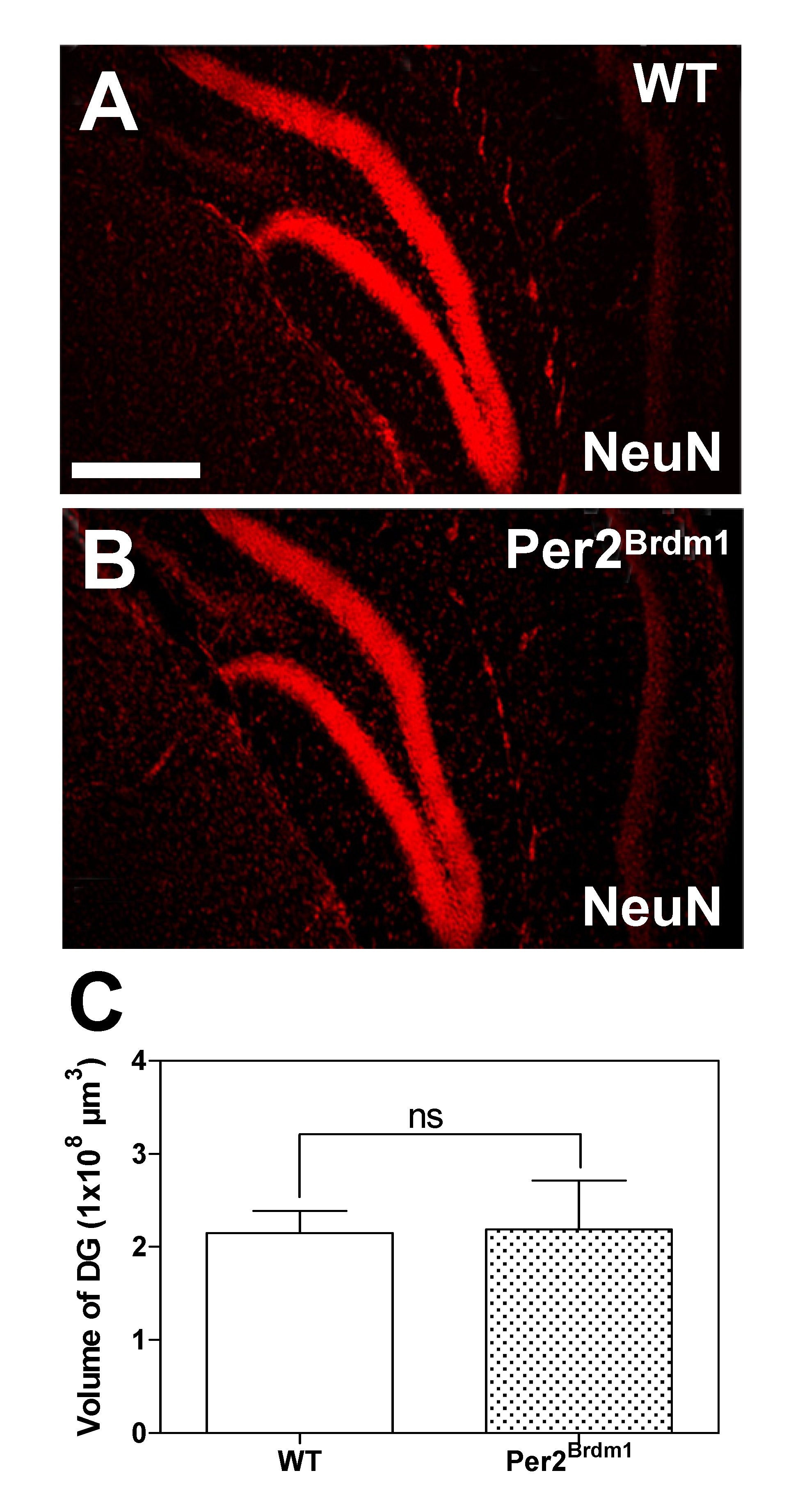

Supplement: Additional file 3 — No significant difference between the volume of the dentate gyrus in WT and Per2Brdm1 mice. (A-B) Confocal images of coronal sections of the DG counterstained with NeuN in order to study the absence of morpho-volumetric differences between WT and mPer2Brdm1 mutant mice at the same level of the rostro-caudal axis. (C) We have compared similar hippocampal areas between WT and mPer2Brdm1 mutant mice and observed that the volume of the granule cell layer (GCL) was not significantly different between the two genotypes (Student's t-test, n = 3, p > 0,05). Scale bar in A = 300 μm for A-B. [file 1471-2202-10-30-S3.tiff]
